# Teaching Personalized Doctor-Patient Communication with AI: PerTRAIN – a Prototype for Interpersonally Responsive Virtual Patients in Medical Education

**DOI:** 10.5334/pme.2379

**Published:** 2026-05-29

**Authors:** Anna Junga, Ole Hätscher, Jennifer Dabel, Gabriyel Mado, Alberta Ajani, Leon Pielage, Pascal Kockwelp, Simon M. Breil, Helena Baur, Jan Siebenbrock, Benjamin Risse, Tanja Grammer, Bernhard Marschall, Mitja D. Back

**Affiliations:** 1 Institute of Education and Student Affairs, University of Münster, Münster, Germany; 2 Department of Psychology, University of Münster, Münster, Germany; 3 Institute of General Practice and Family Medicine, University of Münster, Germany; 4 Institute for Geoinformatics & Institute for Computer Science, University of Münster, Münster, Germany; 5 Joint Institute for Individualisation in a Changing Environment (JICE), University of Münster and Bielefeld University, Germany; 6 University of Münster, Germany

## Abstract

Personalized medicine requires physicians to adapt clinical communication and decision-making to patients’ individual motives, emotions, and interpersonal behaviors. However, training these skills remains challenging, as established simulation-based formats—particularly actor-based simulations—are resource-intensive and difficult to scale. Consequently, there is a need for scalable and flexible training approaches that allow repeated practice across diverse patient personalities and clinical contexts.

The PerTRAIN (Personalization Training in Medicine) project addresses this need by leveraging large language models (LLMs) to simulate virtual patients with dynamically adapting personality expression at scale. Grounded in Contemporary Integrative Interpersonal Theory (CIIT), patient behavior is modelled along the dimensions of agency and communion and updated in response to the medical trainee’s behavior, enabling systematic variation and dynamic adaptation of interpersonal behavior within medical scenarios. This allows trainees to learn how a patients personality shapes communication, and to practice adaptive, patient-centered communication strategies and appropriate clinical decision-making.

For the personalization trainings we developed clinical cases in which personality expression substantially influences behavior in doctor–patient interactions, that represent common encounters in primary care, and that align with national guidelines for patient-centered care. An initial chat-based implementation enables structured interactions, dynamic personality adaptation, and iterative refinement. The system is designed as a complementary tool to existing simulation formats, offering a scalable, low-threshold environment for repeated practice and reflection. A first application in curricular teaching is scheduled for 2026. Future extensions of the framework are discussed, such as large-scale empirical validation, modelling long-term interpersonal trajectories, and the extension of interactions to multimodal formats.

## Background & Need for Innovation

Medical education is increasingly faced with the challenge of preparing students for complex clinical situations that require not only specialist knowledge, but also communication skills, empathy, and patient-centered decision-making. With the advent of personalization in medicine, these aspects are becoming increasingly important, as clinical decisions must be made on a more individualized basis and in dialogue with patients who differ in their interpersonal motives, emotions and behaviors [1,2,3,4,5].

Effective clinical communication relies not only on medical expertise but also on the social skills that enable doctors to handle the wide variety of challenging interpersonal situations encountered in day-to-day clinical practice [6]. Importantly, this includes the skills to recognize different patient personalities (simplified: “How dominant/submissive and friendly/cold is the patient”) and respond appropriately to their individual needs, fears, emotional sensitivities, and interpersonal behavior styles. That is, in real consultations, physicians deal with different patients (with different personalities) and the doctor’s personalized actions are decisive when it comes to information sharing, trust building and adherence.

Currently, clinical communication skills are taught predominantly using Simulation-based learning (SBL), in which clinical, communicative skills are taught within realistic clinical scenarios [7,8,9,10]. Actor-based simulations using simulated patients (SPs) have long served as the gold standard for training such skills (e.g., empathy, persuasiveness) [11]. In these simulations, trainees can engage in realistic consultations across a wide range of clinical and interpersonal contexts. Yet, such formats are resource-intensive and costly [12,13] which makes it difficult to offer trainees many different cases, as well as to give them the opportunity to go through the same cases more often. Thus, there is an urgent need for scalable, interactive, and personalized training formats [13].

Digital training approaches are increasingly adopted as cost-efficient, scalable, and repeatable alternatives to classical actor-based simulations [14,15]. However, they often still fall short in realism, expressiveness, and behavioral variability compared to actual human interaction behavior [16]. Accordingly, there is a need for training approaches that integrate the scalability and standardization of digital methods with the interpersonal realism of actor-based simulations. Recent advances in artificial intelligence (AI), specifically large language models (LLMs), offer a promising avenue for closing this gap. Accordingly, various training systems have been developed recently that already employ LLM-based virtual patients to support communication training (e.g., Yu et al. 2025 [17]). However, in these approaches, the patient’s personality is implemented as a static default that is designed to remain constant throughout the interaction. This limits realism, as real social interactions are inherently dynamic, and patients’ behavior typically changes in response to the clinician’s communication style. For example, if a clinician behaves in an unfriendly or dismissive manner, real patients tend to respond with less cooperative or less friendly behavior themselves. In contrast, a virtual patient with a static personality may continue behaving in a friendly and cooperative way regardless of the clinician’s behavior, thereby oversimplifying the interpersonal dynamics of the interaction, making the training scenario less realistic.

The PerTRAIN project (Personalization Training in Medicine; pertrain.ai) aims to address this limitation by developing more realistic virtual patient simulations. Grounded in interpersonal personality theory [5,17], we leverage LLM-based virtual agents to simulate realistic patient personas across a range of patient personality configurations in a highly standardized and scalable manner. Importantly, rather than treating patient personality as static, our approach models personality expression as a dynamic process that unfolds during the interaction and adapts to the trainee’s behavior. This enables medical trainees to practice recognizing individual differences between patients in symptom description, communication patterns, and medication adherence, to adapt their behavior accordingly, and to reflect on the impact of their actions on clinical outcomes.

## Goal of Innovation

The overarching goal of the project is to develop scalable, LLM-based training that enables medical trainees to practice communication skills across a wide range of clinical contexts, while systematically varying the patient’s interpersonal behavior. These trainings are designed to be adaptable to multiple medical specialties and concrete case types (e.g., chronic disease management, shared decision-making, counselling under uncertainty), allowing for variation in both medical context and patient personality that meaningfully influence interaction dynamics and outcomes.

The core idea of PerTRAIN is to steer LLM-based virtual patients to simulate patients with different personality configurations and to dynamically adapt their interpersonal behavior during the clinical scenario based on the perceived behavior of the medical trainee. The patients differ not only in their typical behavioral tendencies but also in how they respond to the trainee’s behavior over the course of the clinical scenario. Trainees can thus encounter entirely different interpersonal dynamics, experiment with communication strategies, receive immediate feedback, and reflect on essential social skills such as adaptability and empathy, skills that are difficult to train at scale and central to personalization in medicine. This feature enables them to learn how to recognize different patient personalities, adapt their own behavior accordingly, and understand how these adaptations shape outcomes in frequently occurring clinical encounters.

To implement personalization trainings at scale, a diverse set of validated clinical cases based on frequently occurring medical encounters is required. A key conceptual consideration during case design is the deliberate selection of scenarios in which patient personality does not only alter communication style, but also shifts patient priorities, goals, and expectations in clinically relevant ways. Trainees must therefore identify these underlying motives and needs, respond flexibly, and balance them with medical recommendations and economic considerations.

As an illustrative example, consider a common outpatient scenario involving a patient with chronic cardiovascular disease who experiences a medication-related side effect that affects quality of life. While the biomedical problem remains identical across encounters, differences in patient personality are expected to substantially alter priorities, decision-making, and communication.

*Fixed case description*: Middle aged, Caucasian male patient with cardiac disease. Got a prescription for β-adrenergic receptor antagonists, developed erectile dysfunction and discontinues the medication without consultation. He schedules an appointment without stating a specific reason and does not immediately disclose the underlying concern.

Based on this identical clinical background, the system can instantiate distinct patient personas that differ in their interpersonal behavior in the doctor patient interaction, for example:

- **Reserved patient:** Visibly uncomfortable and embarrassed, provides minimal information and is reluctant to disclose details, anxious about cardiac health
- **Unfriendly patient:** Questions the physician’s competence, expresses dissatisfaction with the treatment, refuses medication
- **Demanding patient:** Insists on e.g. MRI before restarting medication, despite lack of indication; proposes alternative treatments

The goal of the personalization training is that trainees recognize these personality differences, adapt their communication strategies accordingly and integrate patient preferences with evidence-based medical decision-making to optimize medical outcomes (e.g., disclosure of symptoms, medication adherence, satisfaction of patient).

## Steps taken for Development and Implementation of Innovation

To achieve the above-mentioned goals, we needed to take the following steps: First, select a personality framework that best describes variation in interpersonal behavior in a medical context. Second, find a way to operationalize the differences in interpersonal behavior and their dynamic adjustments computationally, thereby implementing the unfolding of personality differences within social interactions. Third, conceptualize medical cases that are highly relevant and where personality expression has an impact on clinical outcomes, and that align with the guidelines of the German Society for General Medicine and Family Medicine (DEGAM) [18].

**Figure 1:**
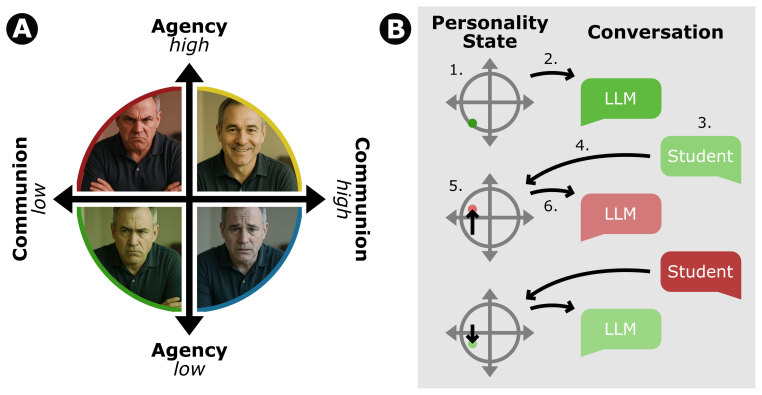
**A** Circumplex adapted from Wiggins 1982, visualization of four prototypical patient profiles aligned with the extremes of the underlying scale. Pictures of Patients were AI-generated using GPT-5 **B** Illustration of continuing state changes depending on conversation.

We drew on Contemporary Integrative Interpersonal Theory (CIIT) [19], one well-established framework for analyzing individual differences in social interactions. It offers a robust foundation for understanding the dynamics inherent in interactions between two conversational partners. Interpersonal behavior is organized along two orthogonal dimensions: agency, denoting assertive (vs. submissive) behavior, and communion, denoting warm (vs. unfriendly) behavior. Together, these dimensions form the Interpersonal Circumplex (IPC), a two-dimensional model that accounts for a substantial proportion of variance in interpersonal behavior (see Figure 1A, adapted from Wiggins [20]). Applied to the examples above, the unfriendly patient would be characterized by low communion, the reserved patient by low agency, and the demanding patient by high agency.

Importantly, an individual’s personality differs not only in how agentic or communal they typically behave (i.e., their behavioral defaults) but also shapes the extent to which they react to the interpersonal behavior of their interaction partner (i.e., their interpersonal reactivities; [21]). Accordingly, the virtual patients were designed to differ in both their behavioral defaults (e.g., high communion combined with high agency resulting in friendly yet dominant behavior) and in their interpersonal reactivities (e.g., dominant behavior by the trainee eliciting dominant responses in some patients and submissive responses in others). Thus, behavioral adjustments over the course of the interaction will be influenced not only by the LLM-based patients’ personality but also by the behavior of the trainee. For example, if the student is very unfriendly the LLM will react accordingly (see Figure 1B).

An initial prototype system has already been implemented by the computer science team, enabling interaction and behavioral adjustments based on the conversation. This system was also used to compare the capabilities and limitations of various current LLMs to identify appropriate models in terms of data privacy, language availability, response speed, and cost. The first version of our LLM-based agent modelling is text-based and comprised of two LLM-based components: one generates patient’s behavior aligned with the conversational content and the current discrete IPC location, while the other analyzes the interpersonal content of the trainee’s messages. For the analysis, an LLM is instructed to generate a score per dimension corresponding to the number of states; this can then be used to increase or decrease the probability of transitioning to that state when selecting the next state. The factor by which the probabilities change, remain constant, or shift toward a standard distribution is configurable. Consequently, the interpersonal behavior of the patient is updated based on the preceding behavior, the partners behavior, and key interpersonal phenomena (complementarity, behavioral defaults, interpersonal reactivities, see Hätscher et al., 2025 for details [20]). The number of states is determined in advance for each IPC dimension, and state transitions are independent of one another for each dimension. A combination of the current states is used for generations.

Medical cases were developed based on the following criteria: (1) We preselected medical encounters in which we expected personality differences to substantially influence key variables (e.g., symptom disclosure, communication style, and medication adherence). (2) We prioritized encounters that reflect genuine consultation patterns seen in everyday primary care, such that the resulting personalization trainings are broadly applicable (e.g., common reasons for consultation in general practice). (3) Case development followed a multi-step process. First, cases were identified based on epidemiological frequency data to ensure that the scenarios reflect common and clinically relevant presentations in general practice [22,23]. The selected cases were then aligned with the learning objectives of the existing curriculum. In a subsequent step, clinical experts in general practice and psychologists reviewed the cases to assess their suitability for representing different personality-related interaction dynamics within consultations. Finally, all cases were aligned with current recommendations of the DEGAM guidelines on the initial anamnesis interview in general practice, which promotes patient-centered communication through active listening, empathy, and exploration of patients’ illness perceptions, expectations, and resources [18]. The anamnesis interview further highlights psychosocial and biographical factors, coping strategies, and shared goal setting, culminating in diagnostic planning, follow-up, and reflective documentation to ensure continuity and communication quality. Afterwards the cases were scripted by medical experts and theatre education specialists.

The chosen reasons for the consultation were based on frequency and relevance: Digestive problems, erectile dysfunction, dizziness, and arterial hypertension. Erectile dysfunction was chosen as a common side effect of arterial hypertension medication [24].

## Outcomes of Innovation

Capitalizing on a unique combination of psychological, data science, and medical education expertise, our integrated outcomes include

1. an evidence-based conceptual framework for varying interpersonal behavior in doctor-patient interactions within relevant medical contexts
2. an innovative method that uses LLMs to dynamically adjust patient personalities over the course of doctor patient interactions
3. initial chat-based prototypes with relevant medical contexts

In the following section, we focus on the development of medical cases and on how personality differences translate into differences in medical communication, as this aspect has the most direct implications for medical education (for more theoretical and technical details on the methodology, see Hätscher et al., 2025 [21]).

The first scenario, titled *“The Uncomfortable Consultation”*, focused on erectile dysfunction. The patient (approximately 50 years old, male, with a history of cardiac disease) had developed erectile dysfunction after starting β-adrenergic receptor antagonists. Because of this and because he did not perceive any immediate benefit from the medication, he had stopped taking it on his own. Aware that this was not a healthy decision, he now presents to his general practitioner. Depending on the patient’s personality (i.e., their behavioral defaults and interpersonal reactivities), he behaves and responds differently during the conversation. A detailed role description was created analogous to the scheme used in the local SP program and was reviewed and refined by the experienced SP trainer team.

The following table details how four prototypical combinations of interpersonal behavior (see Table 1) translate into distinct communication patterns, as well as the specific challenges they pose and the corresponding optimal strategies for the trainee. Case 1 serves as an illustrative example of the development criteria applied to the remaining medical cases.

**Table 1:** Exemplary overview of General Communication and Challenges, Identifying the Problem, and Compliance for the four different basic state combinations.


GENERAL COMMUNICATION AND CHALLENGES		

SP PERSONALITY COMBINATION	COMMUNICATION	PROFESSIONAL CHALLENGE

Agency high, Communion high *(dominant & friendly)*	Open, cooperative, but with clear expectations and straight communication	Maintain a balance between participation and medical guidance – do not allow yourself to be overruled by the “friendly expert” in the patient

Agency high, Communion low *(dominant & cold/confrontative)*	Directive, critical, suspicious, possibly aggressive	Conversations can quickly become confrontational; high risk of resistance or escalation

Agency low, Communion high *(submissive & friendly)*	Calm, polite, avoids confrontation, rarely addresses problems on his own initiative	Conversation seems harmonious, but there is a risk of false compliance; important issues remain unsaid

Agency low, Communion low *(passive & cold)*	Short-tempered, defensive, reserved, deflecting	Difficult to access, high risk of conversation breakdown or avoidance of important topics

**IDENTIFYING THE PROBLEM**		

**COMBINATION (AGENCY/COMMUNION)**	**COMMUNICATION PATH**	**PROFESSIONAL NEEDS**

high/high	Patient actively names symptoms, even embarrassing ones, but selectively and with a focus on solutions	Ask specific questions, verify priorities and psychological stress to identify blind spots

high/low	Patient selectively brings up topics, is skeptical about medical questions, avoids weaknesses	Focusing on facts and emphasizing benefits is required

low/high	Reveals little, but responds openly when asked; requires active exploration	Gentle, structured questioning of potential problems is necessary to uncover hidden issues (such as ED)

low/low	Rarely gives information voluntarily, avoids contact and openness	Laborious, requires a high degree of structure, patience, and dominant conversation steering

**COMPLIANCE**		

**COMBINATION (AGENCY/COMMUNION)**	**ASSUMED COMPLIANCE**	**SPECIFICS**

high/high	High, if convinced, wants to have a say, feels responsible	Shared decision-making is crucial; clear professional communication is necessary

high/low	Low, questions authority, makes their own decisions	Compliance often only occurs with self-motivation – highlight personal goals

low/high	Moderate, happy to cooperate, but without real conviction	Often does not understand the reason, does things “because the doctor says so” – education and participation are important

low/low	Low – avoids, forgets, or ignores therapy	Requires structured follow-up care, continuous motivation, relationship building


**Figure 2:**
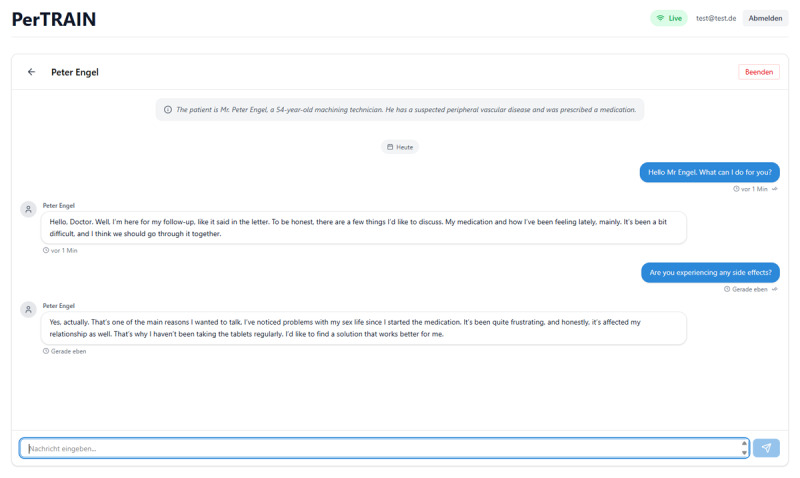
Screenshot of the browser-based application, showing a chat with a sample patient. Shown is the exemplary start of the conversation with Mr. P. Engel.

The complete case vignette for Case 1, as an example of the cases developed, can be found in Appendix 1. Figure 2 shows the view for students in which the cases are processed.

In addition to case 1, further cases were developed: “The Cold”, “The Uneasy Stomach”, “The stressful dizziness”, “The better physician” and “The escape into sick leave”. The other cases are not published in this paper, as their content is to be used in courses. They are available from the authors upon reasonable request.

Within the course, all students will work on the same cases with different types of personalities. Afterwards a structured debriefing by family medicine experts will be provided. These experts can use (graphical and otherwise processed) exports from the virtual conversation for the debriefing.

## Critical Reflection

The development of *PerTRAIN* has been accompanied by continuous reflection on conceptual, methodological, and ethical aspects.

The current text-based prototype serves as an important first step and proof of concept, but its limitations are evident. Here, we focus on three limitations that we are going to adjust in the future, namely (1) the integration of multimodal data, (2) the thorough evaluation with empirical studies, and (3) modelling not only short-term but also long-term interpersonal dynamics.

(1) The virtual patients developed in this work are exclusively chat-based. Consequently, nonverbal elements such as tone, gesture, and facial expression are absent, which raises the question of how well students can perceive and respond to emotional nuances in a purely textual environment. However, recent studies suggest that it is indeed possible to convey and elicit emotional states through chat-based communication alone, indicating that emotional engagement is not entirely dependent on nonverbal cues [25]. At the same time, this constraint was intentional: the chat-based format provides a controlled environment in which linguistic indicators of agency and communion can be isolated and validated before introducing the additional complexity of AI-generated speech with a clearly defined position within the IPC. However, technological advancements in the near future may help to overcome this need, including more realistic and emotionally rich voice-based AI systems, advances in immersive VR simulations, and the integration of these components. Therefore, in the current state it is quite simple for medical experts to add new cases as there is no additional graphical content needed. As there is no “Authoring tool” additional cases must be implemented in cooperation with the technical experts.

(2) Although the medical cases were developed top-down and initially validated by medical experts, large-scale empirical evaluation with trainees (e.g., medical students) is still pending. Accordingly, our next step is to test whether trainees can reliably recognize differences in interpersonal behavior, whether they adapt their communication strategies accordingly, and whether these skills improve across repeated training encounters. Addressing this will require reliable measures to quantify interpersonal trajectories and interaction outcomes across diverse medical contexts and patient personality configurations.

To assess these outcomes, future studies will combine several evaluation approaches. First, interaction logs generated during the simulations will be analyzed to quantify interpersonal trajectories and communication patterns across different patient personality configurations. Second, learner performance will be assessed using structured rating procedures (e.g., expert ratings of communication strategies and situation-appropriate responses). Third, learner-related outcomes such as perceived realism, usability, and perceived learning benefit will be captured through standardized questionnaires and qualitative feedback.

Moreover, we will implement instructional supports to facilitate situation-appropriate adaptation, including AI-generated feedback on communication patterns, real-time visualizations of interpersonal trajectories, and targeted micro-interventions during the interaction. Finally, future work should directly compare the effectiveness of the chat-based prototype with actor-based simulations, with the explicit aim of complementing, rather than replacing, these established training approaches.

(3) Currently, we analyze only short-term interactions (i.e., a single consultation) and the virtual patients do not remember prior interactions with the trainee (e.g. the day before) yet. However, optimal adaptation in doctor–patient communication may depend not only on momentary interpersonal dynamics but also on the accumulated interaction history across prior encounters. Thus, a key extension of our framework involves modelling longitudinal, personalized learning trajectories where trainees can follow the same virtual patient over multiple sessions, observing how the simulated individual’s attitude or trust evolves over time in response to prior interactions. Such continuity mirrors real clinical relationships but is rarely feasible in SPs or VR-based formats due to high personnel and logistical demands.

Looking ahead, the cases developed in this work will be implemented for the first time in curricular teaching in SS26 using the chat-based format. This initial deployment will be accompanied by a user-centered evaluation, which will inform the iterative refinement of the interaction design and the further development of available interaction options.

In summary, PerTRAIN illustrates how LLM-driven patient personas can be used to train communication skills in realistic medical encounters in which trainees engage with diverse attitudes, needs, and interpersonal behaviors. This approach offers a scalable and resource-efficient complement to traditional actor-based simulations, enabling repeated practice and contributing to greater personalization in medical care. Next steps include large-scale empirical evaluation, integration of the scenarios into curricular teaching, and extension of the framework to multimodal interaction.

## Additional File

The additional file for this article can be found as follows:

10.5334/pme.2379.s1 Appendix. Case 1 – The Uncomfortable Consultation; long Version.

## Figures and Tables

**Table:** 

AI	Artificial Intelligence
CIIT	Contemporary Integrative Interpersonal Theory
DEGAM	German Society for General Medicine and Family Medicine (German: Deutsche Gesellschaft für Allgemeinmedizin und Familienmedizin)
IPC	Interpersonal Circumplex
LLM	Large Language Models
PerTRAIN	Personalization Training in Medicine
SBL	Simulation-based learning
SPs	Simulated Patients

## Data Availability

The datasets used and analyzed during the current study are available from the corresponding author on reasonable request.
