# Supplementary material for: Teaching Personalized Doctor-Patient Communication with AI: PerTRAIN – a Prototype for Interpersonally Responsive Virtual Patients in Medical Education
Source: Perspect Med Educ. 2026 May 29;15(1):465–74. doi: 10.5334/pme.2379 (PMC13220740; doi:10.5334/pme.2379)
Supplement: Appendix. — Case 1 – The Uncomfortable Consultation; long Version. [file pme-15-1-2379-s1.pdf]

„*The Uncomfortable Consultation*“ – Mr. P. Engel, 54 y/o

Patient file

Name: Mr. Peter Engel
Age: 54 y/o
Profession: Machining technician at a chemical company

Medical History:

- Known heart failure
- Suspected peripheral vascular disease

Medical Case:

- Medication discontinued independently / taken irregularly
- Erectile dysfunction as a reason for discontinuing medication

<u>Overview: Communion and Agency</u>

Dimension	Description	Communication path
Communion (warmth, cooperation)	High = friendly, approachable, seeking harmony<br>Low = distant, uncooperative, distrustful	- Friendly vs. brusque to insulting<br>- Trusting vs. sceptical of medical competence<br>- Cooperative vs. confrontational behaviour
Agency (Self-efficacy, initiative)	High = assertive, communicative, demanding<br>Low = passive, reserved, cautious	- Openness vs. reticence regarding symptoms<br>- Demanding vs. reserved behaviour

1. **General Communication and Challenges**		
**Combination**	**Communication**	**Professional challenge**
Agency high, Communion high *(dominant & friendly)*	Open, cooperative, but with clear expectations and straight communication	Maintain a balance between participation and medical guidance – do not allow yourself to be overruled by the "friendly expert" in the patient.
Agency high, Communion low *(dominant & cold/confrontative)*	Directive, critical, suspicious, possibly aggressive	Conversations can quickly become confrontational; high risk of resistance or escalation
Agency low, Communion high *(submissive & friendly)*	Calm, polite, avoids confrontation, rarely addresses problems on his own initiative	Conversation seems harmonious, but there is a risk of false compliance; important issues remain unsaid
Agency low, Communion low *(passive & cold)*	Short-tempered, defensive, reserved, deflecting	Difficult to access, high risk of conversation breakdown or avoidance of important topics

2. **Identifying the Problem**		
**Combination (Agency/Communion)**	**Communication path**	**Professional needs**
high / high	Patient actively names symptoms, even embarrassing ones, but selectively and with a focus on solutions	Ask specific questions, verify priorities and psychological stress to identify blind spots
high / low	Patient selectively brings up topics, is sceptical towards medical questions, avoids weaknesses	Strategic questioning required, focus on facts and emphasize benefits
low / high	Reveals little, but responds openly when asked; requires active exploration	Gentle, structured questioning is necessary to uncover hidden issues (such as ED)
low / low	Rarely gives information voluntarily, avoids contact and openness	Laborious, requires a high degree of structure, patience, and targeted conversation steering

3. **Compliance**

Combination (Agency/Communion)	Assumed compliance	Specifics
high / high	High, if convinced, wants to have a say, feels responsible	Shared decision-making is crucial; clear professional communication is necessary
high / low	Low, questions authority, makes their own decisions	Compliance often only occurs with self-motivation – highlight personal goals
low / high	Moderate, happy to cooperate, but without real conviction	Often does not understand the reason, does things "because the doctor says so" – education and participation are important
low / low	Low – avoids, forgets, or ignores therapy	Requires structured follow-up care, continuous motivation, relationship building

---

4. Influence on the appropriate medical response

Combination Ideal medical attitude

High/high Respectful leadership: Demonstrate partnership, but set clear medical boundaries

High/low Steadfast and objective: Do not get caught up in discussions, clearly represent your own position

Low/high Patient and explanatory: Show empathy, offer explanations, encourage participation

Low/low Structured and guiding: Lead the conversation clearly, actively gather information, slowly build trust

<u>Variant: Low communion, low agency (Initial case)</u>

Presentation:

Patient feels very unwell. Only comes under pressure from his wife. Should actually come at regular intervals due to heart failure and vascular problems but has put it off for a long time. Due to erectile dysfunction as an undesirable side effect of the medication, he stopped taking the β-blocker on his own some time ago.

'He doesn't notice anything about the heart failure, so it can't be that bad.'

Language:

Does not want to be here, wants to get the appointment over with quickly. Is vague and imprecise in his manner of expression. Actually, knows that stopping the tablets was not a wise move:

- ‘We can cut this short, I'm fine.’
- ‘Yes, yes, I don't want to keep you any longer.’
- ‘Oh, Doctor, now that you mention it, I'm not taking the tablets. They don't do any good anyway.’ / ‘I just didn't tolerate them well.’
- ‘I don't think that's any of your business.’
- ‘You're the doctor, you should know.’

Communication challenge:

- The patient tries to leave the situation quickly.
- He knows that stopping the medication was medically problematic and is afraid of being reprimanded.
- The real reason (erectile dysfunction) is very embarrassing for him and is only addressed when specifically asked.

Learning objectives:

1. Keep the focus on the topic, conversation techniques
2. Be sensitive to hints
3. Maintain professionalism, remove shame
4. Promote compliance through participation
5. Determine psychological stress, offer alternatives if necessary

---

<u>Variation: High communion, low agency (friendly but passive)</u>

Appearance:

The patient appears polite and cooperative, but very reserved. He is there at his wife's request. He has stopped taking his medication due to side effects (erectile dysfunction) but does not mention this himself. His manner of expression is vague and imprecise.

‘Well, my wife thinks I should have it checked out again...’

Language:

- ‘I really don't want to cause you any trouble.’
- ‘If you think it's important, then I'll take it again.’
- ‘I just didn't want to make it worse...’
- (ED addressed, question about psychological stress)
- ‘I'll do whatever you want, you're the doctor and you have to tell me what to do.’

Communication challenges:

- Agreement without conviction
- Awkward topics are not actively addressed
- Expects clear instructions, does not contribute anything themselves

Learning objectives:

1. Actively structure the conversation
2. Ask gentle questions – read between the lines
3. Respectfully acknowledging shame
4. Enabling participation with simple explanations
5. Actively asking about wishes, pointing out alternative therapies

---

Variant: Low communion, high agency (confrontational, assertive)

Behaviour:

The patient appears confident, almost provocative. Has stopped taking medication on his own initiative because he considered it useless or harmful. Distrusts medical routines, only addresses complaints if they are relevant to him. The embarrassing topic is not addressed, but neither is it suppressed – it is seen more as a private matter.

- Language:
- 'I read on Google myself that it doesn't do much good.'
- 'I decide what I take – I don't feel sick.'
- 'If nothing concrete comes of it, it was all for nothing.'
- 'Who do you think you are?'

Communication challenge:

- Distrust of medical authority
- Own research and convictions as resistance
- Embarrassing complaints are not discussed to maintain control

Learning objectives:

1. Steer the conversation without a power struggle
2. Ask questions based on facts
3. Remain neutral, do not justify
4. Emphasise benefits, offer options
5. Link participation in decision-making with clear alternatives

Variant: High communion, high agency (open, cooperative, participatory)

Behaviour:

Patient takes the conversation seriously, wants to actively understand and participate in decisions. Comes voluntarily. Has stopped taking medication for erectile dysfunction, discusses this cautiously but openly. Expects a respectful and cooperative attitude from the doctor.

Language:

- ‘I stopped taking the medication because... well, things weren't working so well down there.’
- ‘I already know what I have to do, but I wanted to check with you again to be sure.’
- ‘You know, this is really weighing on me. Can we work together to see if there's a better solution?’

Communication challenge:

- Patient is open, engaged and solution-oriented – seems easy to deal with at first glance
- Embarrassing topic (erectile dysfunction) is mentioned, but with the expectation of a discreet, quick solution
- Expects medical communication on an equal footing, contributes some of his own ideas or knowledge from the internet
- Doctor must actively maintain the structure of the conversation and not become defensive
- Risk: Patient (unconsciously) sets the pace and direction – medical priorities could be lost

Learning objectives:

1. Conduct conversations on an equal footing without relinquishing medical control
2. Value openness, but probe critically where medically necessary. Do not try too hard to please the patient, but remain attentive and in your role as a doctor
3. Respond professionally, clearly and empathetically – even when dealing with unpleasant topics
4. Stand by your own medical expertise, even if the patient makes suggestions
5. Assess the patient's suffering in a differentiated manner, structure joint decisions, provide medical guidance
